# Mere rhetoric? Using solidarity as a moral guide for deliberations on border closures, border reopenings and travel restrictions in the age of COVID-19

**DOI:** 10.1136/bmjgh-2021-006701

**Published:** 2021-07-16

**Authors:** Diego S Silva, Carly Jackson, Maxwell J Smith

**Affiliations:** 1 Sydney School of Public Health, The University of Sydney, Camperdown, New South Wales, Australia; 2 Dalla Lana School of Public Health, University of Toronto, Toronto, Ontario, Canada; 3 Faculty of Health Sciences, University of Western Ontario, London, Ontario, Canada

**Keywords:** COVID-19, Health policy, Public Health, SARS

**Summary box**

- Solidarity, and the rhetoric of solidarity, is commonly used by world leaders in the context of global efforts to control COVID-19.
- Solidarity is a relational value that means that persons in positions of power stand with others, usually marginalised persons, for the sake of justice.
- Marginalised persons and communities are best positioned to know what is important to them. As such, solidarity means leading *with* governments of low-income and middle-income countries on COVID-19-related matters.
- Therefore, solidarity means that decisions regarding the continued use or easing of international and regional travel restrictions must be made together by high-income, middle-income and low-income countries.

Every government is responsible for, and accountable to, its own people. But Member States can only truly keep their own people safe if they are accountable to each other at the global level.

- Dr. Tedros Adhanom Ghebreyesus, 74th World Health Assembly, 31 May 2021^1^

As we enter the second half of 2021, the global health community continues to be vexed by the following question: what justifies states’ continued use of international travel restrictions, including border closures, during the COVID-19 pandemic? The corollary to this question is: with declining case counts in a handful of countries and more than 2.25 billion vaccines administered globally, at what point, and under what conditions, is the reopening of state borders and the reduction of travel restrictions justified? The answers to these two questions are multifaceted and includes being clear on a country or region’s epidemiology and the legal obligations of states. However, answering these questions must also balance ethical values that are often implicit and underpin these scientific and legal deliberations. Arguably, the primary ethical challenge in this context is upholding the right of sovereign states to protect their residents and reduce the risk of spreading SARS-CoV-2 while respecting the generally accepted right to the free movement of people^2^ and goods across international borders.

Politicians across the world have repeatedly argued that ‘we’re all in this together’ (or other such similar remarks), on both domestic and global stages, with regard to reducing the morbidity and mortality associated with COVID-19 and, in turn, returning to a ‘normal’, prepandemic life. This appeal to solidarity is laudable but empty unless we understand what we mean when we use the term or invoke its spirit. We argue that a more nuanced understanding of solidarity in global health can provide states and intergovernmental organisations with a useful moral compass to guide deliberations about the continued use, and the future easing, of border closures and other travel restrictions. Moreover, describing what solidarity entails could begin to provide an ethical understanding of well-established calls for collaboration and cooperation that exists in international law—for example, Article 2 of the International Covenant on Economic, Social, and Political Rights^3^— that help shape present-day global health and also shape our thinking about borders in the context of pandemics. We require articulating what solidarity is, why solidarity is morally and instrumentally important, and how solidarity can help guide policy decisions about borders, travel restrictions and freedom.

## The rhetoric of solidarity

Solidarity is increasingly invoked by policymakers and politicians in addressing the challenges related to COVID-19, often as it relates to the actions (or inactions) of high-income countries (HICs) toward low-income and-middle-income countries (LMICs). Solidarity has been, and continues to be, commonly invoked by Dr Tedros Adhanom Ghebreyesus, Director General of the WHO, when advocating for sharing COVID-19 vaccines or other resources. However, solidarity is also frequently invoked by elected officials, including heads of states. For example, writing about vaccine equity in an editorial of the *Washington Post*, Justin Trudeau (Prime Minister of Canada), Sahle-Work Zewde (President of Ethiopia), Moon Jae-in (President of South Korea) and several other world leaders noted that ‘Where you live should not determine *whether* you live, and global solidarity is central to saving lives and protecting the economy [emphasis in original]’.^4^ More recently, when speaking about Europe distributing vaccines in Africa, French President Emanuel Macron stated that doing so ‘is first of all a duty of solidarity’.^5^

The importance of noting the rhetorical use of solidarity is twofold: first, to underscore its centrality in global health politics, and second, to suggest that if politicians and other decision-makers invoke the value of solidarity, they ought to be clear about that to which solidarity commits them. In other words, if solidarity is being invoked to communicate moral commitments that ought to guide action, rather than being invoked as mere platitude (and we will charitably reject such a conclusion), we must be clear about what solidarity requires in this context.

## What is solidarity and why is it important?

In practice, solidarity generally denotes actions and costs that persons in positions of power willingly accept to stand with others, usually marginalised persons or groups, for the sake of justice or fairness.^6^ Solidarity is a relational value, which ‘…holds that individuals and communities are bound together, or mutually interdependent, and thus, that personal and collective well‐being are intimately linked’.^7^ It has an intuitive appeal during public health emergencies, including pandemics, given the adage that viruses do not respect borders. COVID-19 has demonstrated what global successes are possible when countries and regions choose to act in concert with each other,^8^ and what global failures come about when we ignore such an imperative.^9^

Critically, solidarity also requires that when persons in positions of power stand with marginalised groups, it is the latter—that is, the marginalised—who lead in whatever activities they have identified as critical for their well-being. This conclusion rests on the notion of epistemic justice,^10^ which means (among other things) giving primacy to the experiences and knowledge of marginalised persons in matters in which they are protagonists. Stated simply, marginalised persons are best equipped to understand what values and objectives should guide actions taken under the guise of solidarity.

Solidarity justifies action in one of two ways, which are not mutually exclusive: first, we should act in solidarity because it is the right thing to do,^11^ that is, for the sake of justice or fairness. Second, we can invoke solidarity in an instrumental manner, as a means of achieving some other important goals that cannot be achieved unless we act collectively.^12^ In the context of COVID-19, acting in a solidaristic manner means taking actions that reduce risks of transmission for ourselves and others and working to ensure the effects of COVID-19 are not disproportionately located among the least advantaged. The reasons are straightforward: arresting transmission promotes everyone’s well-being, and those persons living in HICs will not be truly safe until everyone is safe from COVID-19 (and vice versa).

What solidarity does not tell us is how to uphold and promote it as a value. Acting in solidarity with others sometimes requires acting in potentially counterintuitive ways. At first glance, solidarity might suggest to us that borders should not be closed insofar as this appears to be insular, self-interested thinking. On the other hand, one might argue that solidarity requires closing borders to arrest the transmission of SARS-CoV-2 since it is in everyone’s interests, so long as some key moral conditions are met (see further).

## Solidarity and coordination on vaccines and borders

The reopening of borders and travel, and the continued use of intermittent international travel restrictions, requires coordinated efforts regionally and globally. There is a growing chorus of states, intergovernmental organisations^13^ (eg, WHO, the World Bank Group, World Trade Organisation, etc) and scholars^14^ calling for countries to work together, particularly on the challenge of equitable vaccine distribution and vaccination passports, which taken in concert are seen as central to reopening international travel. Through the Digital Documentation of COVID-19 Certificates specifications, the WHO ‘…will guide Member States on how to digitally document COVID-19 vaccination status, SARS-CoV-2 test results, and COVID-19 recovery status’.^15^ The push for such coordination is not merely from countries and United Nations agencies, but also encompasses industry, too. For example, the International Air Transport Association (IATA), a trade association for airlines globally, makes it clear that international standards in testing and vaccine harmonisation (ie, ‘globally standardised approach… with regard to equivalent treatment of different vaccines and mutual recognition of vaccination certificates’ between countries) are two necessary conditions for large-scale air travel as COVID-19 begins to wane.^16^ Critically for IATA, they claim that the aviation industry is ready to collaborate in earnest with national governments, ‘… provided governments set benchmarks against which we can plan for restart’.^15^

The instrumental impetus behind efforts to coordinate equitable vaccine distribution and passports for the mass reopening of international travel is intuitively clear, that is, first and foremost, to improve global, regional and local economies. It can also be defended by appealing to the value of solidarity. As noted previously, solidarity acknowledges the relational nature of persons and communities and calls for working together to tackle challenges that cannot be resolved otherwise.

Critically, and perhaps overlooked by politicians and others in positions of power who—nonetheless—invoke the rhetoric of solidarity, acting solidaristically commits the economically and politically powerful to support marginalised persons in leading efforts toward their own empowerment while providing the material resources necessary to ensure everyone’s well-being is promoted and ensured.

In the context of global cooperation in reopening international travel, solidarity means that G7 and G20 nations, as well as intergovernmental organisations, must not make decisions unilaterally without genuine intervention by governments in LMICs and in accordance with their best interests. The colonial history of international health is littered with examples of initiatives being done onto peoples in LMICs. Solidarity is antithetical to such actions. Concretely, representatives from LMICs must have some meaningful decision-making powers when negotiating not just global vaccine distribution (which they do not have, to date) but also on matters of vaccination passports and the movement of persons and goods, more broadly. In short, cooperation between states, as well as between states and the private sector, should not exclude meaningful participation of LMICs.

If we take heads of states seriously when they appeal to solidarity in the context of COVID-19, they must accept the obligations entailed by solidarity as persons in positions of power. In the context of opening borders and reducing travel restrictions, this means working together across countries and between regions to ensure actions taken to ease travel restrictions do not disproportionately negatively impact LMICs. However, more importantly, it also means *leading with* governments from LMICs. Anything less would mean that invocations of solidarity are insincere and hypocritical.

## Data Availability

There are no data in this work.
